# A Bayesian network meta-analysis of three different surgical procedures for the treatment of humeral shaft fractures

**DOI:** 10.1097/MD.0000000000005464

**Published:** 2016-12-23

**Authors:** Hao Qiu, Zhihui Wei, Yuting Liu, Jing Dong, Xin Zhou, Liangjun Yin, Minhua Zhang, Minpeng Lu

**Affiliations:** a Department of Orthopaedic Surgery, Yongchuan Hospital of Chongqing Medical University; b Department of Orthopaedic Surgery, The Children's Hospital of Chongqing Medical University; c Department of Endocrinology, The Second Affiliated Hospital of Chongqing Medical University; d Department of Endocrinology, Yongchuan Hospital of Chongqing Medical University; e Department of Orthopaedic Surgery, The Second Affiliated Hospital of Chongqing Medical University, Chongqing, China.

**Keywords:** humeral shaft fracture, intramedullary nailing, minimally invasive plate osteosynthesis, network meta-analysis, open reduction and plate osteosynthesis

## Abstract

**Background:**

The optimal surgical procedure for humeral shaft fractures remains a matter of debate. We aimed to establish the optimum procedure by performing a Bayesian network meta-analysis.

**Methods:**

PubMed, EMBASE, the Cochrane Library, and Medline were searched for both randomized controlled trials and prospective studies of surgical treatment for humeral shaft fractures. The quality of the included studies was assessed according to the Cochrane Collaboration's “Risk of bias”.

**Results:**

Seventeen RCTs or prospective studies were included in the meta-analysis. The pooled results showed that the occurrence rate of radial nerve injury was lowest for minimally invasive plate osteosynthesis (MIPO; SUCRA probability, 95.1%), followed by open reduction and plate osteosynthesis (ORPO; SUCRA probability, 29.5%), and was highest for intramedullary nailing (IMN; SUCRA probability, 25.4%). The aggregated results of pairwise meta-analysis showed no significant difference in radial nerve injury rate when comparing ORPO versus IMN (OR, 1.92; 95% CI, 0.96 to 3.86), ORPO versus MIPO (OR, 3.38; 95% CI, 0.80 to 14.31), or IMN versus MIPO (OR, 3.19; 95% CI, 0.48 to 21.28). Regarding the nonunion, SUCRA probabilities were 90.5%, 40.2%, and 19.3% for MIPO, ORPO, and IMN, respectively. The aggregated results of a pairwise meta-analysis also showed no significant difference for ORPO versus IMN (OR, 0.83; 95% CI, 0.41 to 1.69), ORPO versus MIPO (OR, 2.42; 95% CI, 0.45 to 12.95), or IMN versus MIPO (OR, 2.49; 95% CI, 0.35 to 17.64).

**Conclusion:**

The current evidence indicates that MIPO is the optimum choice in the treatment of humeral shaft fractures and that ORPO is superior to IMN.

## 1 Introduction

Humeral shaft fractures are common injuries, making up about 1%–3% of all adult fractures.^[1,2]^ Treatment of humeral shaft fractures includes surgery and conservative treatment.^[3]^ Conservative treatment frequently leads to malunion and some complications of prolonged immobilitysuch as shoulder and elbow stiffness.^[3]^ Currently, surgical treatment is the preferred option, with the most common surgical methods being open reduction and plate osteosynthesis (ORPO), intramedullary nail (IMN), and minimally invasive plate osteosynthesis (MIPO).^[4,5]^

Traditional ORPO can achieve anatomical reduction and rigid internal fixation under direct vision, but the surgical trauma is large, easily leading to wound infection or radial nerve injury and other complications.^[6]^ IMN can protect the integrity of the periosteum, reduce soft tissue dissection, and promote fracture healing, but antirotation ability after IMN is poor.^[2,6,7]^ MIPO has the advantages of protecting the broken end blood supply, less trauma, and fewer complications, but it is more difficult to reset.^[8]^ Since each of these 3 different surgical methods has advantages and disadvantages, there is controversy regarding which represents the best surgical approach.

Many clinical trials have been carried out on the different surgical methods for the treatment of humeral shaft fracture. Traditional meta-analysis can only compare 2 different operation modes, and therefore cannot comprehensively evaluate 2 or more interventions, whereas network meta-analysis is a new statistical method which can be used to compare multiple methods.

Therefore, we performed a Bayesian network meta-analysis to provide more useful information about the utility of different surgical interventions for humeral shaft fractures.

## 2 Methods

### 2.1 Search strategy

We conducted a computerized search of the electronic databases PubMed, EMBASE, Cochrane Library, and Medline until the end of January 2016, according to the PRISMA (Preferred Reporting Items for Systematic Reviews and Meta-Analyses), for randomized controlled trials and prospective studies comparing different surgical procedures in the treatment of humeral shaft fractures. The keywords used included “humeral shaft fracture,” “plate,” “intramedullary nail,” “minimally invasive osteosynthesis,” “randomized controlled trials,” and “randomized”. Secondary searches of unpublished literature were conducted in Google Scholar and Medical Matrix until the end of January 2016. The references cited in these articles were also reviewed to identify any additional studies not previously identified in the initial literature search. Our study was approved by the Research Ethics Committee at our institution Committee.

### 2.2 Selection criteria

Studies with the following criteria were included: (1) patients: patients who were diagnosed with humeral shaft fracture were included in the study; (2) intervention: ORPO, IMN, and MIPO; (3) comparisons: comparisons between any 2 of the 3 methods were included; (4) outcomes: radial nerve injury and nonunion; (5) study: randomized controlled trials or prospective studies. The exclusion criteria were as follows: (1) duplicates or multiple publications of the same study, retrospective studies or case reports, and (2) study did not report outcomes of interest.

### 2.3 Quality assessment

The quality of the included studies was independently assessed by 2 reviewers (according to the Cochrane Collaboration's “Risk of bias” and the Newcastle–Ottawa Scale score). The Cochrane Risk of Bias Tool of Review Manager version 5.3 (Copenhagen, Denmark: The Nordic Cochrane Centre, The Cochrane Collaboration) was applied. Appraisal criteria included: random sequence generation, allocation concealment, blinding of participants and personnel, blinding of outcome assessment, incomplete outcome data, selective reporting, and other bias. Each of these factors was recorded as low risk, unclear risk or high risk. Where data were unclear, we contacted authors for clarification, where possible. Disagreements were resolved by third party adjudication.

### 2.4 Data extraction

Two researchers independently extracted and cross-checked data on trials. The decision to include studies was made initially on the basis of the study title and abstract. When a study could not be excluded with certainty at this stage, the full text was obtained for evaluation. Disagreements were resolved by discussion and, where necessary, in consultation with a third reviewer. Extracted information included the first author, publication year, study design, characteristics of participants, and information to assess the risk of bias. If any data were missing from the trial reports, the reviewers attempted to obtain the data by contacting the authors.

### 2.5 Traditional pairwise meta-analysis

The pairwise meta-analysis was performed completely in Stata 13.0 (Stata Corporation, College Station, Texas). For dichotomous variables, odds ratio (OR) with 95% confidence interval (CI) was calculated. For continuous variables, standardized mean difference (SMD) with 95% CI was calculated. The assessment for statistical heterogeneity was calculated using the chi^2^ statistic and *I*^2^ statistic. If there was no heterogeneity (*P* > 0.05, *I*^2^ < 50%), a fixed effects model was used. Otherwise, a random effects model was used. The outcomes for all direct comparisons were reported.

### 2.6 Bayesian network meta-analysis

A Bayesian network meta-analysis is designed to pool direct and indirect or different indirect outcomes simultaneously. WinBUGS version 1.4.3 (MRC Biostatistics Unit, Cambridge, UK) was used for our Bayesian network meta-analysis using Markov chain Monte Carlo (MCMC) methods. To gain convergence, we performed each MCMC chain with 40,000 iterations and 10,000 burn-in. Thin value was 3. We used the graphical tools in Stata 13.0 (Stata Corporation, College Station, Texas) to present the results of statistical analyses in WinBUGS 1.4.3. Radial nerve injury and nonunion were presented as OR with 95% CI. The results were presented using the surface under the cumulative ranking curve (SUCRA). The SUCRA value was presented as the percentage of the area under the curve, and the higher SUCRA value reflected the better treatment method.

### 2.7 Inconsistency analysis

Disagreement between direct and indirect evidence can suggest that the transitivity assumption might not hold. The inconsistency factors in the closed loop were assessed by the method described by Chaimani et al.^[9]^ Inconsistency analysis was presented as a funnel plot.

## 3 Results

### 3.1 Search results

**Figure 1:**
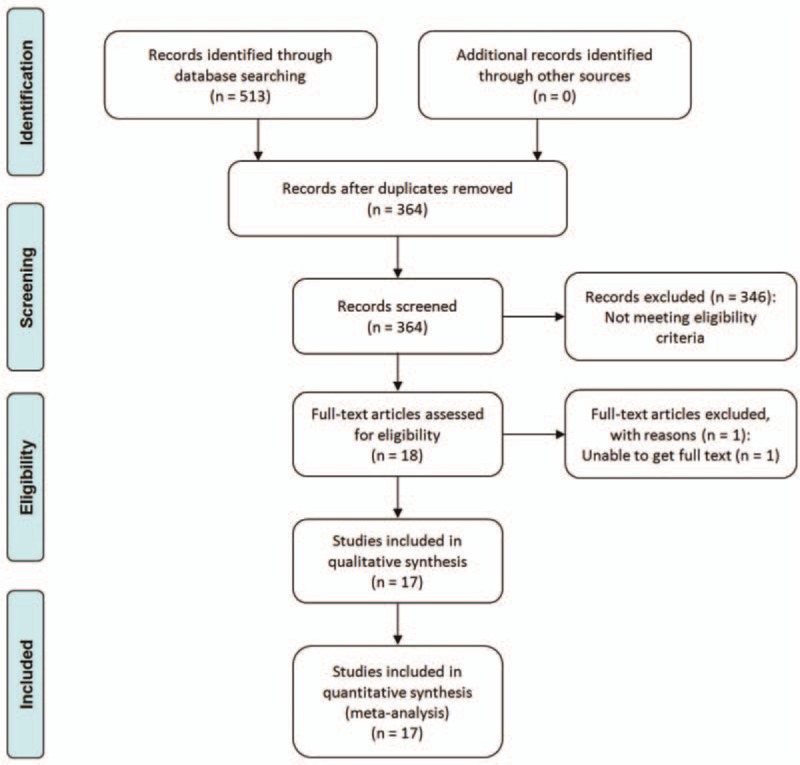
PRISMA 2009 flow diagram. PRISMA = Preferred Reporting Items for Systematic Reviews and Meta-Analyses.

**Figure 2:**
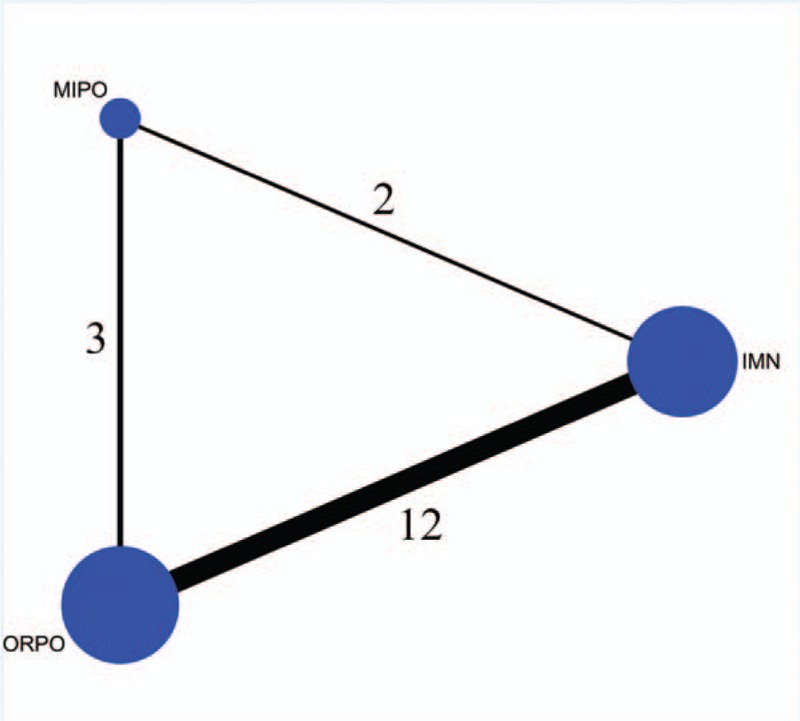
Relationship between the interventions in the network meta-analysis.

A total of 513 records were reviewed; 346 studies were excluded on the initial review of the title and abstract because they clearly did not match our inclusion criteria. After removing duplicates, 149 records were screened. One RCT was excluded because we could not obtain the full text. Finally, 17 RCTs or prospective studies^[10–26]^ met the eligibility criteria and were included in our network meta-analysis. The study selection process and reasons for exclusion are summarized in Fig. 1. The relationship between the interventions in the network meta-analysis is presented in Fig. 2.

### 3.2 Quality assessment and basic information

**Figure 3:**
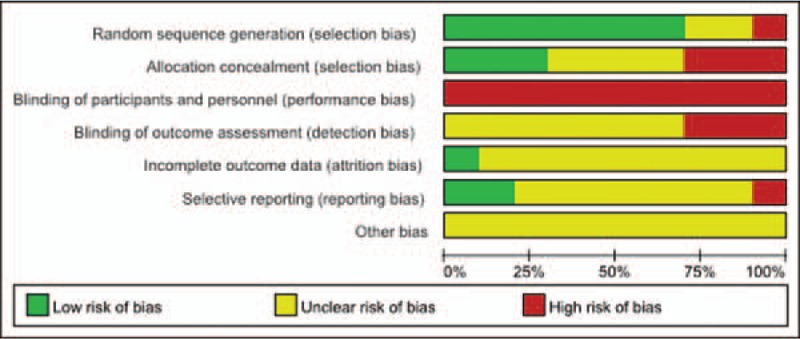
Risk of bias graph.

**Figure 4:**
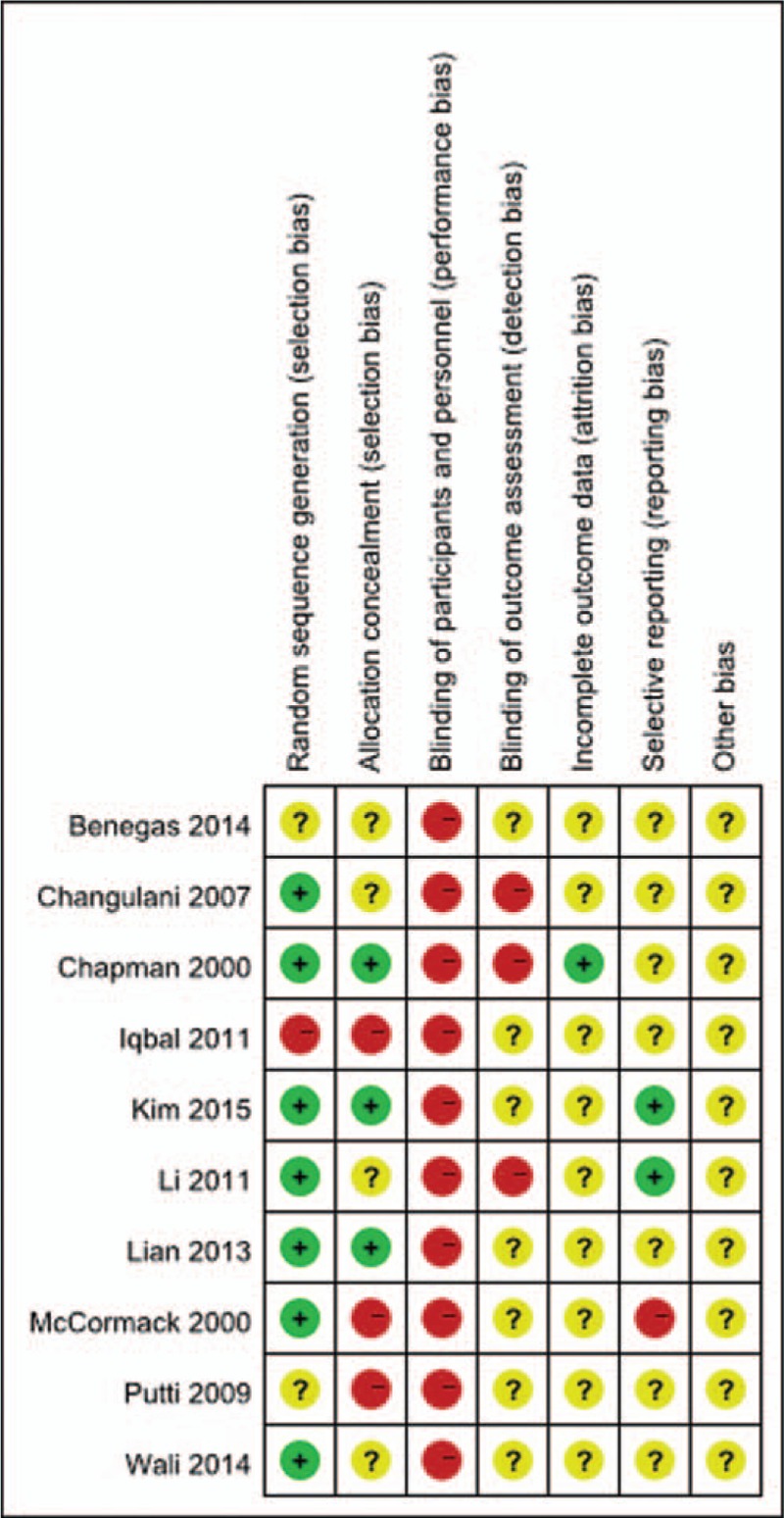
Risk of bias summary.

The quality of the included RCTs was assessed using the Cochrane Collaboration's “Risk of bias.” The risk of bias assessment of included studies is given in Figs. 3 and 4. The risk of bias of the included non-RCTs evaluated with the Newcastle–Ottawa Scale score is demonstrated in Table 1. Ten RCTs^[10,11,13,16,18,19,21–23,25]^ and 7 prospective studies^[12,14,15,17,20,24,26]^ were included, and a summary of their characteristics is presented in Table 2. These studies were published between 2000 and 2015. A total of 815 patients were enrolled in our studies. As described in each study, patients treated by both methods were comparable in terms of gender, side involved, and injury mechanism. All of the studies involved patients with humeral shaft fractures who were followed up for at least 12 months. All the articles evaluated the clinical efficacy of the different surgical procedures in the treatment of humeral shaft fractures. The sample sizes of the included studies ranged from 30 to 84.

**Table 1:**
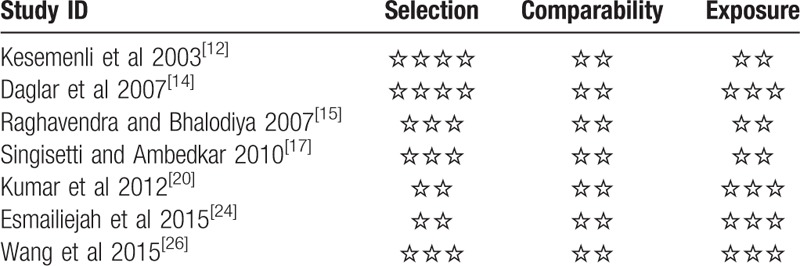
The Newcastle–Ottawa Scale score of non-RCT.

**Table 2:**
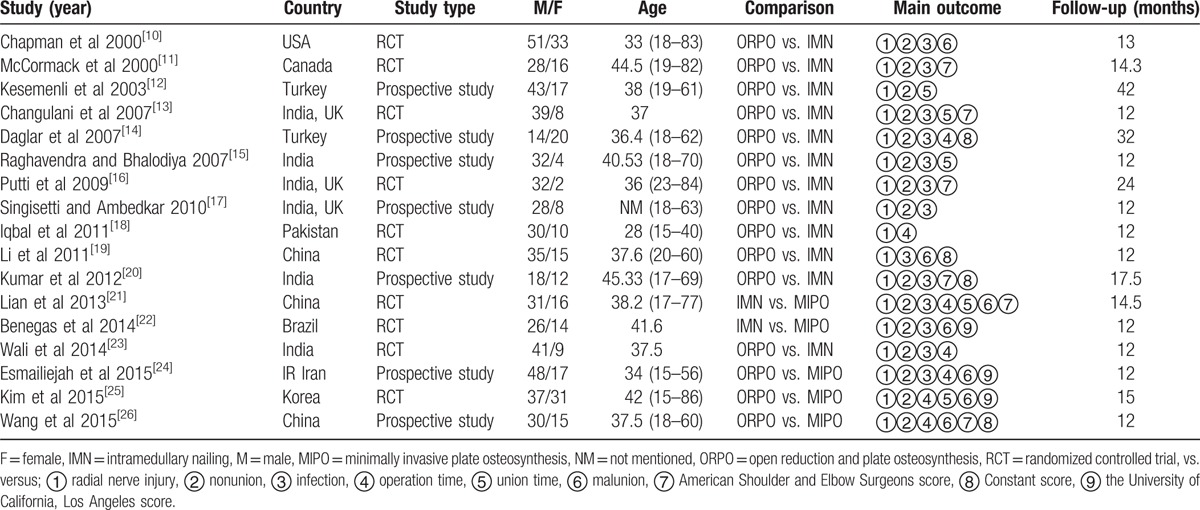
Characteristics of the included studies.

### 3.3 Results of pairwise meta-analysis and network meta-analysis

#### 3.3.1 Radial nerve injury

**Figure 5:**
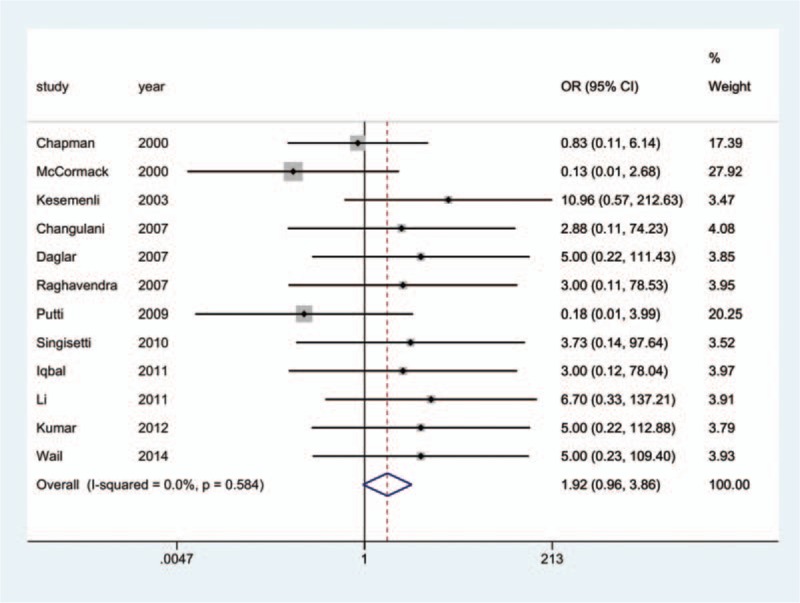
Forest plot of pairwise meta-analysis for radial nerve injury (ORPO vs. IMN). IMN = intramedullary nailing, ORPO = open reduction and plate osteosynthesis.

**Figure 6:**
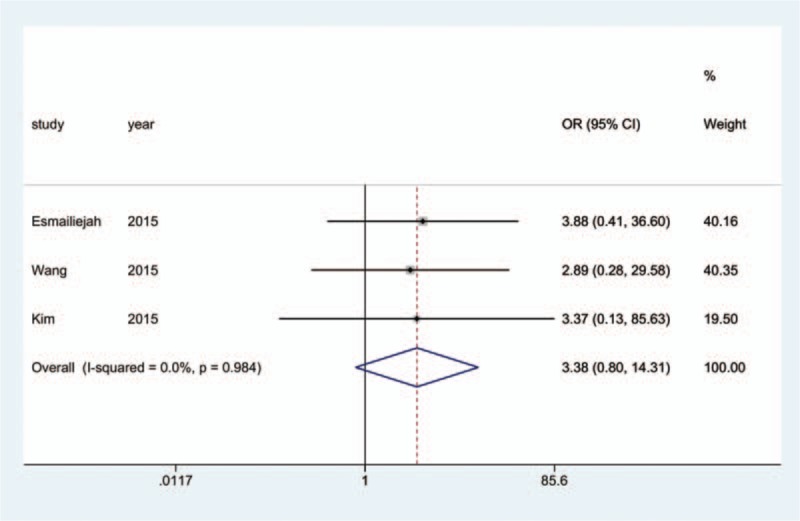
Forest plot of pairwise meta-analysis for radial nerve injury (ORPO vs. MIPO). MIPO = minimally invasive plate osteosynthesis, ORPO = open reduction and plate osteosynthesis.

**Figure 7:**
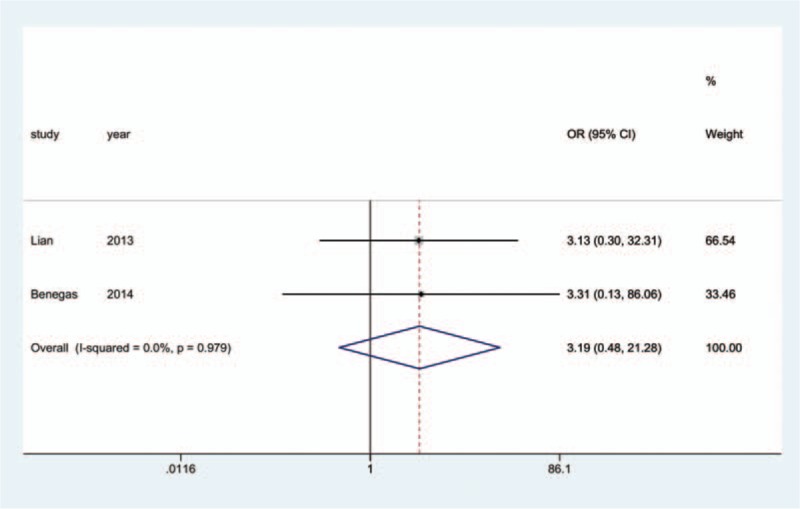
Forest plot of pairwise meta-analysis for radial nerve injury (IMN vs. MIPO). IMN = intramedullary nailing, MIPO = minimally invasive plate osteosynthesis.

**Figure 8:**
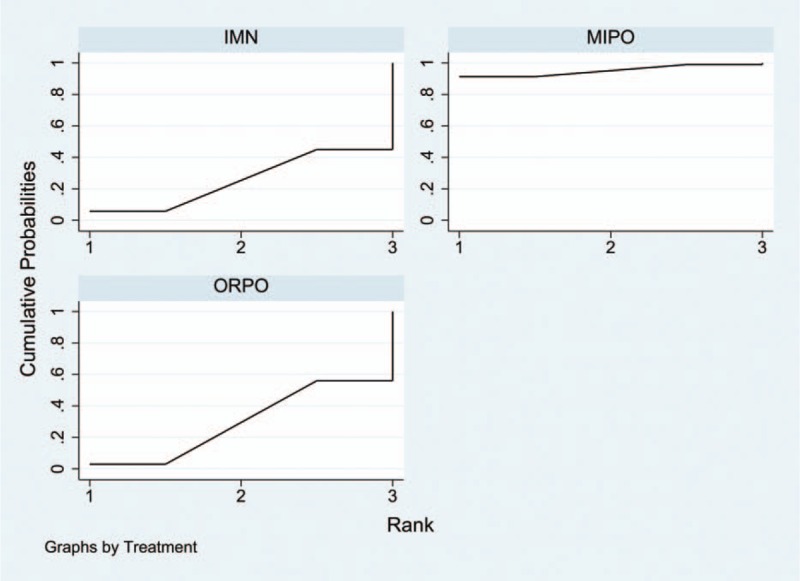
Surface under the cumulative ranking curve for radial nerve injury.

**Figure 9:**
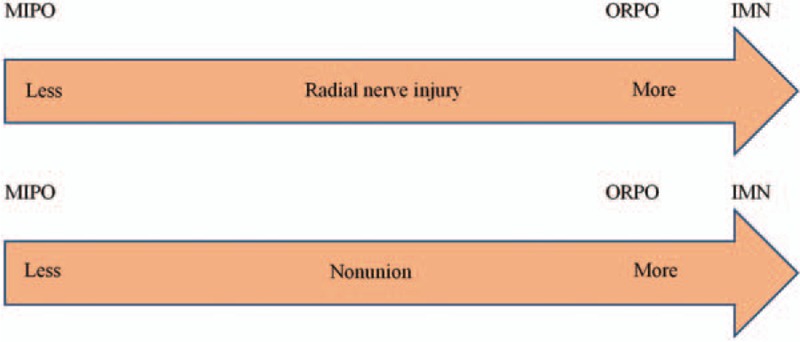
Ranking of treatments in terms of radial nerve injury and nonunion.

Information on the incidence of radial nerve injury was provided in all 17 studies.^[10–26]^ The aggregated results of the pairwise meta-analysis showed no significant difference when comparing ORPO versus IMN (OR: 1.92, 95% CI: 0.96 to 3.86) (Fig. 5), ORPO versus MIPO (OR: 3.38, 95% CI: 0.80 to 14.31) (Fig. 6), and IMN versus MIPO (OR: 3.19, 95% CI: 0.48 to 21.28) (Fig. 7). Similarly, the pooled results of the network meta-analysis showed no significant difference when comparing ORPO versus IMN (OR: 1.44, 95% CI: 0.12 to 6.38), ORPO versus MIPO (OR: 6.34, 95% CI: 0.89 to 26.01), and IMN versus MIPO (OR: 8.82, 95% CI: 0.66 to 43.12). However, SUCRA probabilities were 29.5%, 25.4%, and 95.1% for ORPO, IMN, and MIPO, respectively (Fig. 8). The ranking of the 3 different surgical procedures in terms of the probability of radial nerve injury is shown in Fig. 9.

#### 3.3.2 Nonunion

**Figure 10:**
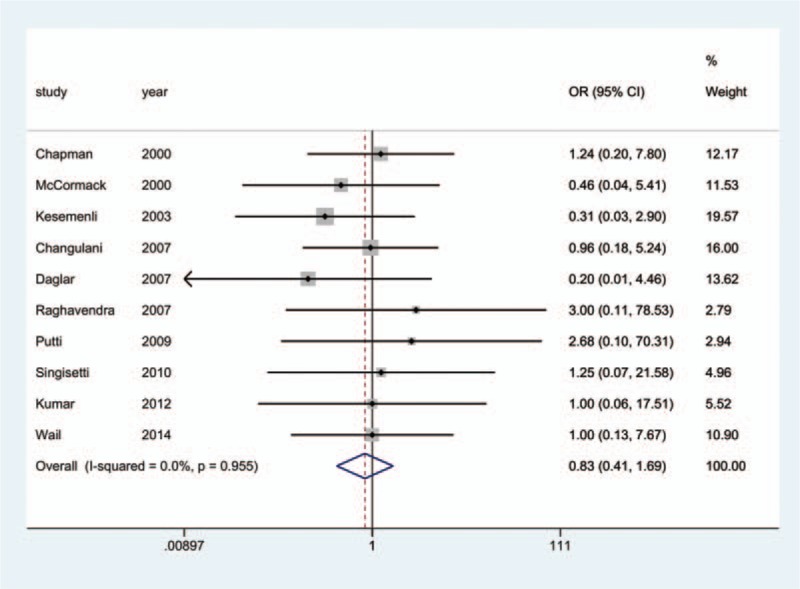
Forest plot of pairwise meta-analysis for nonunion (ORPO vs. IMN). IMN = intramedullary nailing, ORPO = open reduction and plate osteosynthesis.

**Figure 11:**
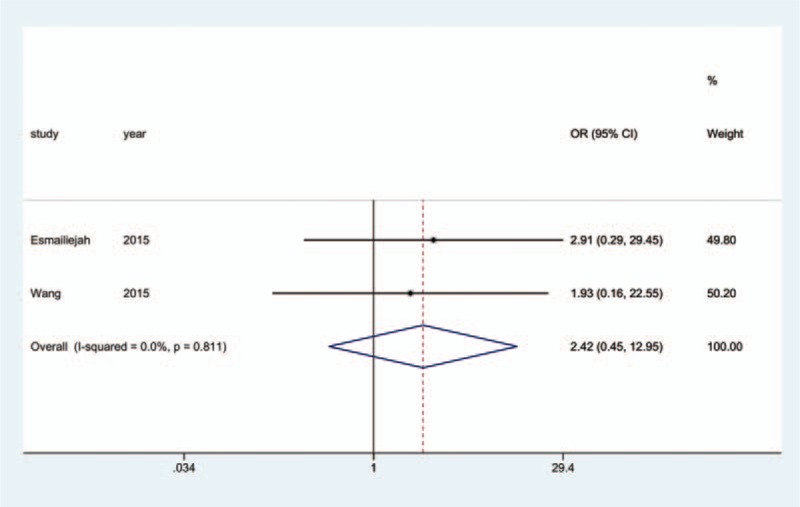
Forest plot of pairwise meta-analysis for nonunion (ORPO vs. MIPO). MIPO = minimally invasive plate osteosynthesis, ORPO = open reduction and plate osteosynthesis.

**Figure 12:**
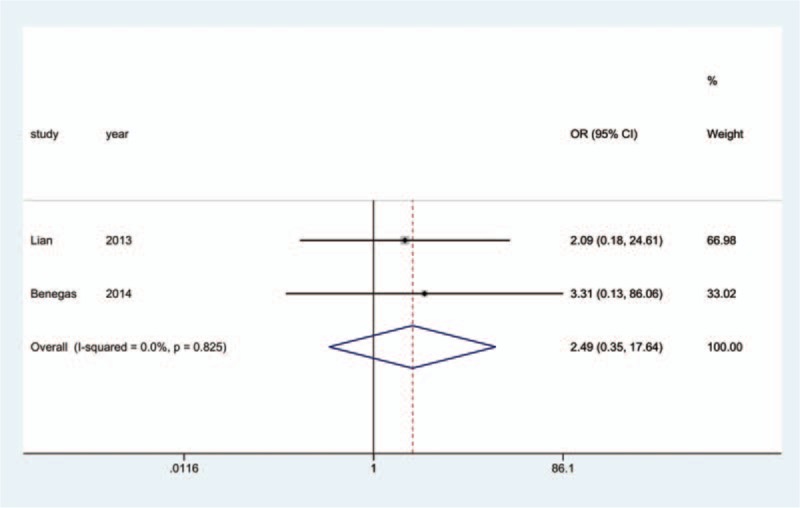
Forest plot of pairwise meta-analysis for nonunion (IMN vs. MIPO). IMN = intramedullary nailing, MIPO = minimally invasive plate osteosynthesis.

**Figure 13:**
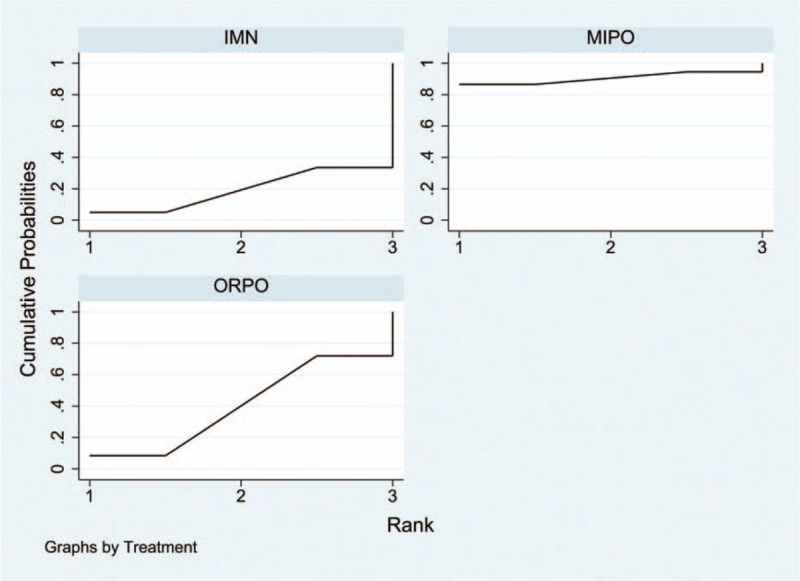
Surface under the cumulative ranking curve for nonunion.

All 17 studies reported the incidence of nonunion; since 3^[18,19,25]^ of the studies reported that no nonunion occurred in any group, we analyzed the data of the remaining14 studies.^[10–17,20–24,26]^ The aggregated results of pairwise meta-analysis showed no significant difference when comparing ORPO versus IMN (OR: 0.83, 95% CI: 0.41 to 1.69) (Fig. 10), ORPO versus MIPO (OR: 2.42, 95% CI: 0.45 to 12.95) (Fig. 11), and IMN versus MIPO (OR: 2.49, 95% CI: 0.35 to 17.64) (Fig. 12). Similarly, the pooled results of the network meta-analysis showed no significant difference when comparing ORPO versus IMN (OR: 0.88, 95% CI: 0.34 to 1.88), ORPO versus MIPO (OR: 3.75, 95% CI: 0.57 to 14.03), and IMN versus MIPO (OR: 4.88, 95% CI: 0.63 to 18.96). However, SUCRA probabilities were 40.2%, 19.3%, and 90.5% for ORPO, IMN, and MIPO, respectively (Fig. 13).

### 3.4 Inconsistency analysis

**Figure 14:**
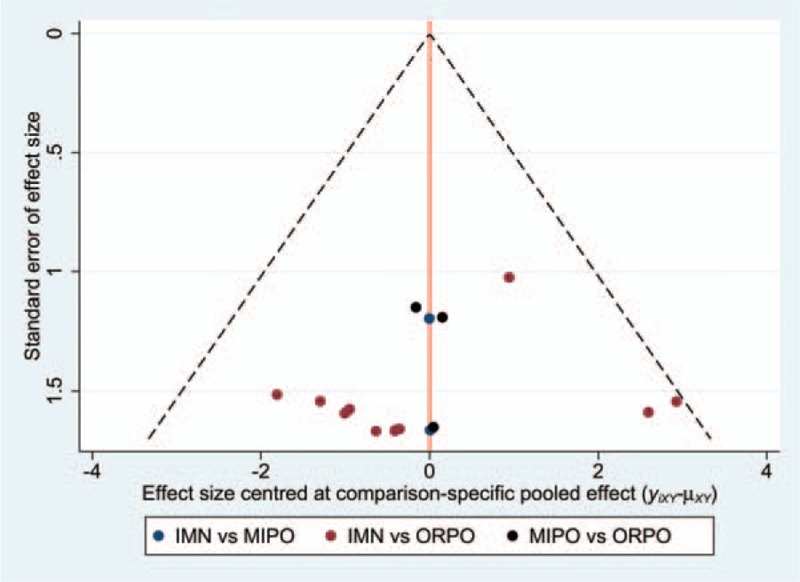
Funnel plot of this Bayesian network meta-analysis.

**Figure 15:**
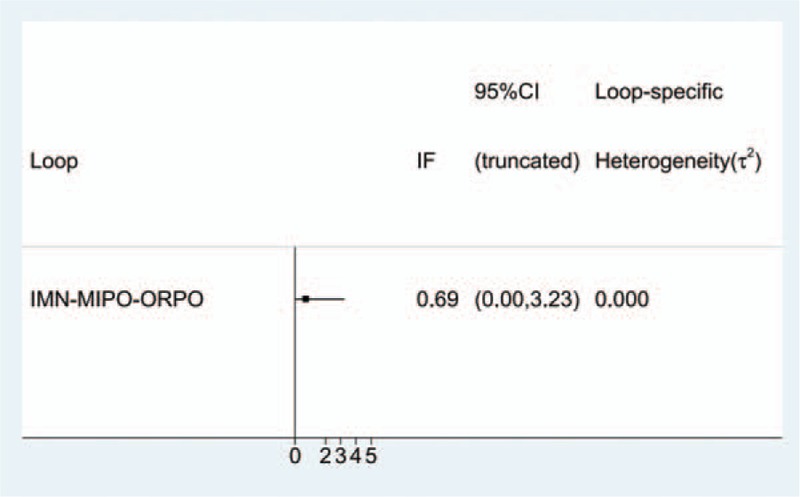
Inconsistency plot of this network meta-analysis.

The funnel plot was symmetrical in general, suggesting that publication bias for the included literature was controlled acceptably (Fig. 14). Inconsistency test results showed an inconsistency factor (IF) of 0.69 (95% CI: 0 to 3.23), which implied that there were no small sample study effects in the closed loop of the network meta-analysis (Fig. 15).

## 4 Discussion

The optimal surgical procedure for humeral shaft fractures remains a matter of debate. Recently, several meta-analyses of the management of humeral shaft fracture have been published,^[27–30]^ but these only focused on the comparison between ORPO and IMN. However, MIPO is playing an increasingly important role in the treatment of humeral shaft fractures.^[21,22,24–26]^ Therefore, we considered it necessary to perform a Bayesian network meta-analysis to compare all 3 methods.

Regarding the incidence of radial nerve injury and nonunion, both the aggregated results of the pairwise meta-analysis and the Bayesian network meta-analysis showed no significant difference between any 2 of the 3 treatments based on OR values. However, the results of SUCRA ranking suggested that MIPO had the lowest probability of radial nerve injury and nonunion than ORPO and IMN. In addition, ORPO had a lower probability of radial nerve injury and nonunion than IMN. Hence, we concluded that MIPO is the optimum choice in the treatment of humeral shaft fractures.

At present, MIPO for humeral shaft fractures involves both an anterior and a posterior approach. Vilaca and Uezumi^[31]^ suggested that the anterior approach is more secure since the plate is placed in front of the humerus in a fixed humeral shaft fracture; the front of the humerus is smoother because there are no major blood vessels and nerves passing through it, and also the anterior approach has the advantage of not needing to reveal the radial nerve. Therefore, it is unlikely to cause iatrogenic radial nerve injury. The studies^[21,22,24–26]^ included in the Bayesian network meta-analysis all used this anterior approach to fix the humeral shaft fracture.

We did not evaluate the postoperative shoulder joint function because the evaluation indicators used in the studies were not uniform. Although most^[11,13,14,16,19,21–23,25]^ of these studies evaluated the shoulder joint function after operation, they used different scoring systems, such as the American Shoulder and Elbow Surgeons (ASES) score, the Constant score, the University of California, Los Angeles (UCLA) shoulder scale, and so on. Some RCTs^[11,13,14,16,19,23]^ have compared the shoulder joint function of ORPO and IMN in the treatment of humeral shaft fracture with different shoulder scores. Their results showed that the effect of ORPO was similar to IMN on postoperative shoulder function. A meta-analysis of 8 RCTs and 2 quasi-RCTs by Ma et al^[28]^ also supports this conclusion. An RCT by Benegas et al^[22]^ evaluated the shoulder joint function after MIPO and IMN for the treatment of humeral shaft fracture, and found no significant difference between the 2 groups (P = 0.98). However, Lian et al^[21]^ suggested that the shoulder joint function score after MIPO was significantly higher than that after IMN (*P* < 0.001). An RCT by Kim et al^[25]^ evaluated shoulder joint function after MIPO and IMN in the treatment of humeral shaft fracture, and found no significant difference between the 2 groups (P = 0.264.). Therefore, we concluded that the 3 different surgical procedures for the treatment of humeral shaft fractures can obtain similar shoulder joint function. But the results of 2 traditional meta-analyses^[27,29]^ showed that IMN may cause more method-related complications and shoulder impingement than ORPO. Therefore, we suggest that ORPO and MIPO are superior to IMN for humeral shaft fractures in regard to shoulder joint function, whereas, on the basis of current evidence, both OPRP and MIPO can achieve a similar treatment effect on humeral shaft fractures.

The present meta-analysis has potential limitations. First, different studies used different inclusion and exclusion criteria and follow-up time, which possibly created some of the heterogeneity we observed among trials. Second, different studies use different evaluation methods, and results of a future comparison would be more convincing if more RCTs use the same evaluation methods. Third, the qualities of the recruited studies were quite different. Some studies demonstrated adequate randomization, but others had incomplete random sequence generation, weak blinding, or imperfect allocation concealment. This limitation might be resolved by an updated Network meta-analysis restricted to high-quality studies, once sufficient become available.

## 5 Conclusion

In summary, our network meta-analysis suggests that MIPO is the optimum choice in the treatment of humeral shaft fractures and that ORPO is superior to IMN. Some traditional meta-analyses^[27–30]^ and systematic reviews^[6,32]^ indicated that both ORPO and IMN can achieve similar fracture union with a similar incidence of radial nerve injury, whereas IMN was associated with an increased risk of shoulder impingement, more restriction of shoulder movement, a higher incidence of implant failure, and so on. They support our conclusion that ORPO is superior to IMN for the treatment of humeral shaft fractures. Due to the limited numbers of patients included in the literature, there is still a need for more well-designed, high-quality studies to further verify this conclusion.
